# The optimal duration for the wrinkle test in a clinical setting

**DOI:** 10.1038/s41598-022-13083-7

**Published:** 2022-05-25

**Authors:** Erez Grinbaum, Ahmad Shahwan, Amir Eliyahu, Ravit Shay, Nimrod Rozen, Guy Rubin

**Affiliations:** 1 Orthopedic Department, Emek Medical Center, Afula, Israel; 2 Faculty of Medicine, Technion, Haifa, Israel

**Keywords:** Neuroscience, Physiology

## Abstract

To determine the time needed or the development of a positive result on the wrinkle test among patients with complete laceration of a digital nerve in a clinical setting. We prospectively recruited 20 patients who had undergone surgery for digital nerve laceration. The wrinkle test was conducted at a follow-up session up to 2 months after surgery, and the time to a positive wrinkling result was recorded. The wrinkle test was compared between the patient’s injured versus uninjured contralateral finger. The average time required for a positive result on the wrinkle test was 24.5 min (± 11), with 25% patients requiring 40 min to obtain a positive result. When evaluating a patient with digital nerve injury in a "non-laboratory" environment, the wrinkle test may require up to 40 min to obtain a positive result. Our study suggests that if tests are completed following the generally accepted time limit reported in the literature (30 min), up to 25% of tests may produce false negative results.

## Introduction

**Figure 1:**
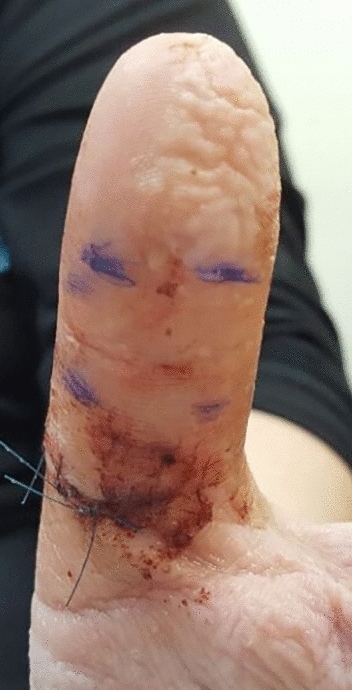
The injured digit distal to the laceration showing no wrinkling while the healthy side is wrinkled.

A penetrating injury of the palm is a common type of injury and may involve the sensory digital nerve. Several tests have been designed to assess the integrity of the digital nerve. In 1973, O'Riain first described the "wrinkle test." This test compares the appearance of skin wrinkles in innervated versus denervated skin after being soaked in warm water for 30 min. O'Riain found that in contrast to skin with intact innervation, denervated skin does not wrinkle after being soaked in warm water^1^. (Fig. 1).

Another study compared the wrinkle test to the static 2-point discrimination in patients after a complete or partial laceration of the median/ulnar/digital nerves and found a good correlation between the tests for complete lacerations but not for partial lacerations^2^. The test has been found to correspond to autonomic function in patients when used at the bedside^3–7^. Previous studies have reported that the optimal water temperature for the test is 40 °C^8^ and that tonicity also plays a role, with hypertonic solutions slowing the time to wrinkling^9^.

The physiology of the wrinkling response includes vasoconstriction because of sympathetic activation^3,7,10–14^. It is widely assumed that the wrinkling affect appears after 5–30 min of soaking in warm water in healthy individuals^12,14^.

Our previous research has shown the wrinkle test to be the most sensitive test for evaluation of digital nerve integrity following finger laceration^15^. In general, past studies evaluating the skin wrinkling test have largely been conducted in a controlled environment. In this study, we sought to evaluate the skin wrinkle test, particularly the time needed to achieve a positive result in a real-life setting.

## Methods and materials

This prospective study (one sample with self-controls) was approved by the Emek medical center review bord, all the patients sign an inform consent, all methods were performed in accordance with the relevant guidelines and regulations. The study population included those underwent surgery for digital nerve laceration (Intervention) and were eligible to the inclusion/exclusion study’s criteria. As control, the patient’s injured finger was compared to the contralateral uninjured finger during the repeated wrinkle tests, until reaching matched, positive outcome. The dependent variable is the positive outcome of the injured finger in the wrinkle test, and the independent variable is the post-wrinkle-test time until the outcome is positive.

**Figure 2:**
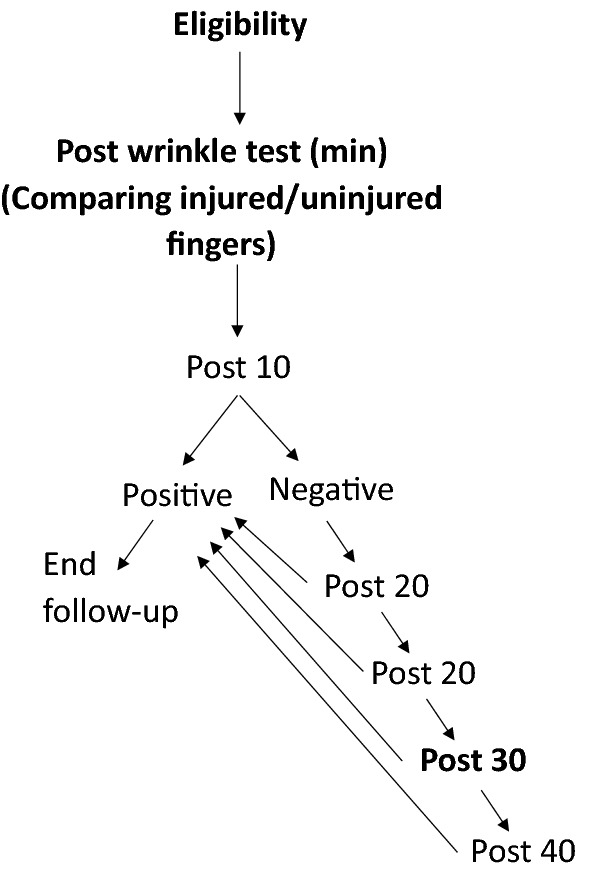
Research flow chart.

We recruited 20 patients who underwent surgery for digital nerve laceration in our department. Patients under the age of 18 years, legally incompetent patients, patients with a history of previous nerve injury, and patients with a known neuropathy were excluded from the study. The power of the study was calculated (as withing subject control) to be at least 80%. The wrinkle test was conducted at a follow-up session up to 2 months after surgery. Research flow is depicted in Fig. 2.

In the clinic, patients were instructed to soak their hands in a 40 °C water bath measured with a thermometer at the start of the study. Every 10 min, the skin of the injured finger was visually compared to the skin of the uninjured finger on the contralateral side until the clinician confirmed a definite positive or negative result for the injured digit. The clinician were trained using the wrinkle assessment scale^16^.

To analyze results, we used IBM SPSS Statistics v24. We used a one sample t-test for comparing the different post-wrinkle-test times to the common (criterion) 30 min timepoint.

### Ethical approval

Ethical approval to report these cases was obtained from the Emek medical center review bord (APPROVAL NUMBER/0134-18-EMC).

### Informed consent

Written informed consent was obtained from the patient(s) for their anonymized information to be published in this article. The authors have read the Journal's position on issues involved in ethical publication.

## Results

The study included 20 patients (6 female and 14 male) with an average age 42 years (range, 21–64 years). All patients had experienced the complete laceration of a digital nerve, and were otherwise healthy, without background any morbidity or chronic pharmacologic treatments.

The test was carried out an average of 21 days after surgery (range, 8–30 days). Table 1 lists details regarding the affected hand and finger, side of the digital nerve, zone of injury, and dominant hand. The average time to get a positive result (Table 1) was 24.5 min (range, 10–40 min). When compared to the 30-min wrinkling time, which is the acceptable time in the literature, our sample had a significantly shorter average time to a positive result, *t*(11) = 2.238, *P* < 0.037.

**Table 1:** Patient's demographics, injury characteristics and results.

Number	Age	Gender	Dominant hand	Injured hand	Injured finger	Flexor zone	Digital nerve side	Time from operation (days)	Optimal duration for the wrinkle test (minutes)
1	23	Male	Right	Left	2	2	Radial	22	20
2	52	Female	Right	Left	2	2	Ulnar	26	20
3	54	Female	Right	Left	4	2	Radial	20	40
4	51	Male	Right	Right	2	1	Radial	18	30
5	41	Male	Right	Right	2	2	Radial	19	10
6	40	Male	Right	Right	2	2	Radial	14	30
7	30	Male	Right	Left	2	1	Radial	20	20
8	44	Male	Right	Left	1	3	Ulnar	24	40
9	35	Female	Right	Left	1	2	Radial	21	40
10	52	Male	Left	Right	5	2	Ulnar	20	20
11	64	Male	Right	Left	2	2	Ulnar	22	40
12	55	Female	Right	Left	4	3	Radial	30	10
13	21	Male	Right	Left	4	1	Radial	27	20
14	24	Male	Right	Left	3	3	Ulnar	29	20
15	44	Male	Right	Left	5	2	Ulnar	22	30
16	21	Male	Right	Right	2	1	Ulnar	19	10
17	49	Male	Right	Right	5	2	Radial	23	40
18	58	Female	Right	Left	5	2	Radial	18	20
19	53	Male	Right	Left	4	2	Ulnar	8	20
20	27	Female	Right	Right	4	1	Radial	10	10

Of note, four out of the 20 patients (20%) showed wrinkling after 10 min, eight patients (40%) showed wrinkling after 20 min, three patients (15%) showed wrinkling after 30 min, and five patients (25%) showed wrinkling after 40 min.

## Discussion

The wrinkle test has been prescribed for both pre-^13^ and post-surgical^1^ evaluation for digital nerve laceration, and the generally accepted timeframe for the test has been stated as 30 min^1^. The current study was designed to evaluate the optimal time for the wrinkle test in a clinical setting and determine whether it may be necessary to extend the test time to avoid false negative results.

We found that 25% of the patients were positive only after 40 min. One possible reason for the longer required time may be the cooling of the water during the test and the difficulty in maintaining the water at 40 °C over the course of the test. A study that examined the ideal water temperature in healthy people showed that a temperature of 40 °C leads to skin wrinkling in 3.5 min, while the use of colder water extends the time required for skin wrinkling^8^.

Another possible reason for the longer duration required to interpret a positive result could be the difficulty in assessing the presence or absence of wrinkles. In a previous study, we demonstrated that a skin wrinkle test, which is the only objective test among other tests assayed, was the most sensitive test to detect a digital nerve laceration pre-surgery. However, the test was challenging to interpret in approximately 20% of the cases^15^.

In this study, we found interpreting the accepted scale for skin wrinkle can be difficult when comparing the injured and uninjured hands with digits that experienced penetrating trauma. We demonstrated that with a 30-min wrinkling time as the acceptable time in the literature, we demonstrated a standard deviation of 11 min. Another possible reason for the longer time needed for positive result and for the difficulty interpreting the result can be the callous of the patient's hand due to heavy manual labor.

The sensitivity and specificity of the skin wrinkle test has been tested in previous studies. In cases of polyneuropathy, the sensitivity and specificity were found to be 71.4% and 73%, respectively, and the positive and negative predictive values were 83.3% and 57.4%, respectively^16^. Another study examining the test in patients with neuropathy of the small nerves in the foot found the sensitivity and specificity to be 66% and 70%, respectively^17^. This assessment was not performed when testing patients with a peripheral nerve laceration in the hand.

Alternatives and modifications for the skin wrinkle test have also been tested. A previous study reported that aqueous solutions of differing tonicity led to different results for time to wrinkling and the best results were found in hypotonic water^9^. In addition, applying the eutectic mixture of a local anesthetic cream has been reported to provide results like the water immersion technique^11,16^.

The main limitation of this study was the relatively small number of patients included. Nonetheless, even in this group, we were able to see variation in the time to a positive result, with a large proportion of the population needing longer than the accepted timeframe for a definitive result. This supports the study hypothesis that for cases of known digital nerve laceration, the test time needed to provide a reliable result can be up to 40 min under standard clinical conditions. Nevertheless, this test is the only objective test for nerve laceration and should be included in our clinical investigation of a patient with suspected nerve laceration.
